# Novel *LAMA2* Gene Mutations Associated with Merosin-Deficient Congenital Muscular Dystrophy

**DOI:** 10.29252/.22.6.408

**Published:** 2018-11

**Authors:** Feyzollah Hashemi-Gorji, Vahid Reza Yassaee, Parisa Dashti, Mohammad Miryounesi

**Affiliations:** 1 Genomic Research Center, Shahid Beheshti University of Medical Sciences, Tehran, Iran; 2 Immunology, Asthma and Allergy Research Institute, Tehran University of Medical Sciences, Tehran, Iran

**Keywords:** Creatine Kinase, Genetic counseling, Mutation, Reverse transcriptase polymerase chain reaction

## Abstract

**Background:**

Merosin-deficient congenital muscular dystrophy (MDC1A) is a rare autosomal recessive genetic disease occurred due to mutations in the *LAMA2* gene. This study investigated the molecular genetics of three Iranian MDC1A patients who manifested hypotonia, muscle weakness at birth, elevated levels of creatine kinase, and normal magnetic resonance imaging before the age of six months.

**Methods:**

Peripheral blood samples were collected from three unrelated patients and their families after obtaining informed written consents. Genomic DNA was extracted and sequenced using next-generation sequencing, followed by Sanger confirmation.

**Results:**

Sequencing results revealed a known missense mutation, c.8665G>A, and two novel heterozygous sequencing variants affecting splicing, c.397-4_c.478del and c.7452-1G>A, in the *LAMA2* gene. Reverse transcriptase-PCR analysis showed that a new intronic variant, c.7452-1G>A, produced aberrant splicing pattern in the patient.

**Conclusion:**

This study expands the mutation spectrum of *LAMA2* and assists in the diagnosis, genetic counseling, and prenatal diagnosis of the affected families.

## INTRODUCTION

Merosin-deficient congenital muscular dystrophy (MDCMD, CMD, MDC1A) is a rare autosomal recessive genetic disease involving the central and peripheral nervous system in the childhood (OMIM: 607855)[1]. MDC1A is common in China and Western countries[2,3]. Affected infants present with muscle weakness, hypotonia, failure to thrive, poor suck and cry, and delayed motor development[4,5]. A previous study has shown a correlation between the levels of merosin expression and clinical severity[6]. The disorder is caused by the mutations in the *LAMA2* (Gene ID: 3908, OMIM: 156225) on chromosome 6q22, which encodes the α2 chain subunit of laminin 2 (merosin) and laminin 4 (s-merosin)[7]. A recessive mutation in this gene also causes a wide phenotypic spectrum of *LAMA2*-related muscular dystrophy from a severe early-onset CMD to a mild later childhood-onset limb-girdle type muscular dystrophy[8-10]. Signs and symptoms in early-onset *LAMA2*-related muscular dystrophy (early-onset *LAMA2* MD) appears at birth or within the first few months of life, including contractures of the large joints, profound hypotonia, poor spontaneous movement, and severe muscle weakness. Diagnosis of early-onset *LAMA2* MD is based on clinical examination, high serum creatine kinase (CK) concentrations, merosin deficiency detected by immunohistochemical staining of muscle or skin biopsy, and white matter changes on brain MRI. Clinical manifestation in late-onset *LAMA2* MD are similar to a group of muscle disorders classified as limb-girdle muscular dystrophies and are milder than in the early-onset type. Late-onset *LAMA2* MD has clinical overlap with Emery-Dreifuss myopathy due to elbow contractures, high serum CK concentrations, and prominent spinal rigidity; however, major cardiac involvement is absent in *LAMA2* MD[11]. Muscular dystrophies are a group of heterogeneous disorders that have overlapping clinical symptoms, which may lead to misdiagnosis. The efficiency of the next-generation sequencing (NGS) had previously been reported for the molecular diagnosis of the CMD, based on clinical and laboratory findings[12-14]. It has been shown that NGS-targeted panels of 40 genes can be a useful approach to identify causative variants in CMDs[13]. However, this method is not cost-effective when there is a clinical overlap. A previous report from Iran showed an improved diagnostic yield of neuromuscular disorders applying clinical exome sequencing[14]. In this study, we aimed to investigate the molecular genetics of three unrelated Iranian MDC1A patients using whole exome sequencing (WES) method, followed by data analysis of known CMD genes.

## MATERIALS AND METHODS

This study was approved by the Ethics Committee of the Deputy of Research Affairs, Shahid Beheshti University of Medical Sciences (Tehran, Iran). Three patients who participated in this study were from unrelated Iranian families upon previous diagnosis/suspicion of merosin deficiency. Blood samples were collected from the patients and the parents after obtaining their informed written consents. Genomic DNA was extracted using conventional salting-out protocol[15]. WES was performed by Macrogen Inc. (Seoul, Korea) and sequenced on illumine HiSeq 4000 at the mean coverage of 100×. The DNA samples were prepared according to an Agilent SureSelect Target Enrichment Kit preparation guide. The SureSelect Target Enrichment workflow was used to capture the regions of interest, enriching them out of an NGS genomic fragment library guide, followed by paired-end sequencing. The sequencing read was aligned and mapped to hg19 from UCSC Genome Browser (https://genome.ucsc.edu), and the variants were annotated and filtered using a custom bioinformatics pipeline for *LAMA2* gene in first step and 62 genes in the next step, as previously described in detail[14]. In this study, variants with minimum allele frequency (MAF > 0.001) were excluded. Only, rare variants (MAF < 0.001 or new) with nonsense, nonsynonymous, splice site, and insertion and deletion variants in known or related genes to merosin deficiency disorders were selected for interpretation.

Identified pathogenic variants were confirmed in the patients and segregated within the families using specific primer pairs and Sanger sequencing (Table 1). For analysis of splicing mutation, cDNAs were synthesized using PrimeScript RT reagent kit (TAKARA, Japan) after the extraction of total RNAs from peripheral blood using TRIzol® reageant (Qiagen, USA) following the manufacturer’s protocol. Then the partial-length *LAMA2* cDNA was amplified by reverse transcription-PCR (RT-PCR) using one specific primer pair cDNA-F and cDNA-R (Table 1), followed by 2.5% agarose gel electrophoresis. All primers and sequencing results were designed and analyzed using Gene Runner 6.2.07-Beta and Chromas 2.4, respectively. The mutations nomenclature was based on the cDNA nucleotide position, according to the *LAMA2* genomic sequence in the GenBank database (NM_000426.3) and Mutalyzer 2.0.21 (https://www.mutalyzer.nl) (Table 2).

**Table 1:** List of primers and PCR condition for confirmation of mutations

Primer name	Primer sequence (5’ to 3’)	Annealing temperature (°C)	Extension (s)	Product size (bp)
LAMA2-F3	TCAGGTGAAATGTTGCCAATGAG	60	60	571
LAMA2-R3	TTTCTGACAGGCCTATTTCACCG			
LAMA2-F54	GAACATCCATTTAGACCAACCAG	58	60	507
LAMA2-R54	TGGATCACAATTCTAGGACTTC			
LAMA2-F61	AGACTTCGACCTAAAACTGACC	58	60	523
LAMA2-R61	TGACTTCCTATTCACCTATCAG			
cDNA-F	ATATAGCAACTTCGTCTTCTGG	58	40	318-330
cDNA-R	TCTTGGTGCTGAATGACAGGT			

**Table 2:** A summary of mutation identified in the *LAMA2* gene

Patient	Nucleic acid alternation	Amino acid alternation	Location	Zygosity	Affected domain	GenBank accession No.	ClinVar accession
1	c.8665G>A	Gly2889Arg	Exon61	Hom.	LamG4	KY054725	SCV000323176.1
2	c.397-4_c.478del	-	Intron3_Exon4	cHet.	LamG2	KY100117	SCV000590914.1
	c.7452-1G>A	-	Intron53	cHet.	LamNT	KY100118	SCV000590915.1
3	c.8665G>A	Gly2889Arg	Exon61	Hom.	LamG4		

Hom, homozygous; Het, heterozygous; cHet, compound heterozygous; Lam, laminin; LamNT, laminin N-terminal domain

Bioinformatics tools were used to test the pathogenicity of the mutations, including PolyPhen-2 (http://genetics.bwh.harvard.edu/pph2/), SIFT (http://sift.jcvi.org), Mutation Taster (http://www.mutationtaster.org), UMD-Predictor (http://umd-predictor.eu), and Combined Annotation Dependent Depletion (CADD) (http://cadd.gs.washington.edu/)[16-20]. In addition, the protein modeling was performed based on the PDB 5IK7, as a template, to construct a model for the mutation analysis using SWISS-MODEL[21,22].

## RESULTS

### Clinical description

Patient 1, a seven-year-old boy, who belonged to a consanguineous couple, was born by normal delivery. His mother experienced pregnancy with intrauterine fetal death. Initial symptoms arisen in him since birth included muscle weakness, inactivity, and hypotonia. At the age of ten months, concentrations of lactate dehydrogenase, CK, and aldolase were found to be 2005 IU/l (normal range 180–430 U/L), 1248 U/l (normal range 200-400 U/l), and 13.9 IU/l, respectively. Electromyography (EMG) results showed low amplitude and duration of the motor unit action potential (MUAP). The CK and aldolase levels were measured to be 2136 U/l and 12 U/l, respectively, when the patient was two years old. The MRI of the brain was normal at one year of age, and there was no history of seizure. At the age of two years and six months, immunohistochemical (IHC) analysis results showed weak and patchy sarcolemmal labeling with merosin antibody. Muscle biopsy results showed severe myopathic atrophy with endomysial fibrosis, compatible with muscular dystrophy. At age seven, he had kyphosis, short elbow, and contractures of elbow and wrist. The CK and aldolase levels decreased to 523 U/l and 5.5 U/l, respectively (Table 3). He attended a regular school having normal intelligence (with IQ score estimated to be 160). Based on clinical and biochemical finding, MDC1A was proposed.

**Table 3:** A summary of patients under study, including age of onset, biochemical analysis, clinical examination, and muscle biopsy results

Patients	Gender	Age (y)	Age at follow-up (months)	Age of onset	Consanguinity	Prominent symptoms	MRI before the age one	CK (U/l)^#^	LDH (U/l)	Aldolase (U/l)	Muscle biopsy/IHC
1	M	7	10	birth	Yes	Muscle weakness, inactivity, hypotonia, kyphosis	normal	1248	2005	13.9	Merosin positive CMD
2	M	5	5	birth	No	Hypotonia, myopathic face, kyphoscoliosis	normal	1762	871	21.0	Merosin positive CMD
3	M	6	5	birth	Yes	Hypotonia, kyphosis	normal	6304	NA	44.0	Merosin positive CMD

# CK normal range: 200-400 U/l depending on the laboratory[11] .NA, not assigned

Patient 2 was a five-year-old boy who was delivered via caesarean section at full-term and belonged to a non-consanguineous marriage. At the time of birth, the child manifested hypotonia and a myopathic face. There was no history of CMD in the family. At the age of five months, the concentrations of lactate dehydrogenase, CK, and aldolase were found to be 871, 1762, and 21 U/l, respectively. Motor and sensory nerve conduction results were normal with no evidence of peripheral neuropathy. The EMG results showed moderate positive sharp waves, fibrillation, low amplitude, and duration of motor unit activities. The EMG, motor, and sensory conduction analyses proposed a diagnosis of the spinal muscular atrophy, and due to the short duration and low amplitude of the motor unit, occurrence of congenital myopathy was not ruled out in this case. At age three, the genetic test result was negative for *SMN1* gene using the multiplex ligation-dependent probe amplification method. At age four, the result of muscle biopsy analysis was compatible with the muscular dystrophy, and the IHC study of the sarcolemmal proteins showed a loss of labeling of all muscle fibers and nerve bundles with merosin antibody (Table 3). At age five, clinical examination showed kyphoscoliosis and contracture of elbow and wrist. Considering the results, a genetic study of the *LAMA2* gene was suggested.

Patient 3 was a six-year-old boy born into healthy consanguineous parents. He showed hypotonia since birth. The CK and aldolase were found to be 6304 and 44 U/l, respectively. At the age of five months, the result of muscle biopsy and IHC tests demonstrated dystrophic changes. Then MRI study of the brain was suggested for the patient in which was normal at the age of six months. Based on clinical and biochemical finding, merosin deficiency was proposed, and genetic test for the *LAMA2* gene was recommended. At the age of six, he had kyphosis, and cognitive function was normal with no history of seizure (Table 3).

### Sequencing results

The biochemical analysis showed a high level of the serum CK in both patients. The molecular analysis results revealed pathogenic variants in the *LAMA2* gene. In patients 1 and 3, a known missense mutation, c.8665G>A (p.G2889R), was identified, in which was segregated within the families. In patient 2, two novel splice site mutations, c.397-4_c.478del and c.7452-1G>A, were found in *LAMA2*, as a compound heterozygote (Table 2). The segregation analysis in patient 2 revealed that compound heterozygote mutation was in *trans* statues. He had inherited c.397-4_c.478del from his father and c.7452-1G>A from his mother. The frequencies of identified variants are very low in normal population or absent in databases including ExAC, 1000G, dbSNP, gnomAD, Iranome, and an in-house database of nearly 500 WES results. No additional incidental findings were identified in the analysis of other genes related to muscular dystrophy. The result of RT-PCR analysis of c.7452-1G>A mutation in patient 2 showed a different pattern of the *LAMA2* mRNA expression in comparison to his parents and a healthy control. Partial *LAMA2*-cDNA analysis in the patient with heterozygote GA genotype (c.7452-1G>A) demonstrated a reduced *LAMA2*-mRNA expression (318-330 bp) rather than his mother who was also (lane-3) heterozygote for c.7452-1G>A mutation. It may happen due to nonsense-mediated mRNA decay of another allele with c.397-4_c.478del mutation in the patient. The mother had not c.397-4_c.478del mutation, while the father and the patient were heterozygote for this mutation.

## DISCUSSION

In this study, we reported biochemical and genetic data obtained from three Iranian patients with MDC1A (Table 2). The patients in this study had a severe phenotype of MDC1A with normal cognitive function. The patients manifested hypotonia, muscle weakness, high levels of CK, and positive results in the muscle biopsy IHC studies. The absence of laminin α2 antibody in muscle fibers, nerves, and skin biopsy is useful for differential diagnosis of *LAMA2*-related disorder versus αDG-related disorder (Alpha dystroglycan-related dystrophies)[3]. The molecular analyses of the patients identified a known mutation, c.8665G>A (p.G2889R), in two patients and a novel compound heterozygous mutation, including c.397-4_c.478del and c.7452-1G>A mutations (Table 2).

**Fig. 1:**
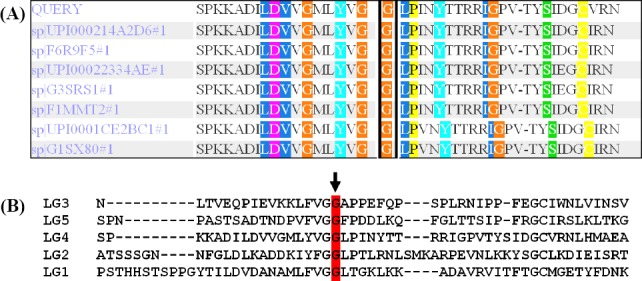
Conservation analysis of amino acid sequences among different species (A) and LG domains (B). The p.G2889R variant site is located in a highly conserved amino acid position among different species (marked with a black box in A). Figure 1B shows the conservation of p.G2889R among LG domains from LG1 to LG5 domains (highlighted in red).

**Fig. 2:**
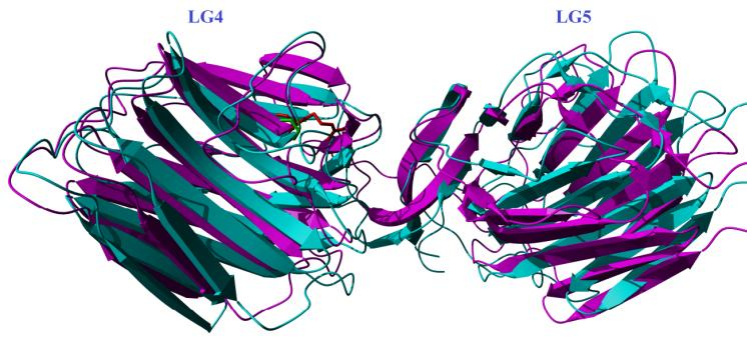
A model of the p.G2889R mutation. Wild type residue (in green) and mutated residue (in red) are shown based on PDB 5IK8.

In this study, c.8665G>A was found in two affected patients with MCDA1 in homozygous state. According to the American College of Medical Genetics and Genomics guideline (ACMG), c.8665G>A variant was first classified as the variant of likely pathogenic mutation in patient 1 and patient 2. This mutation has also been reported for the first time as a pathogenic mutation in an affected patient with MCDA1 in homozygote status[14]. c.8665G>A is a missense mutation that results in the substitution of Arg amino acid at conserved position 2889. Its frequency in population databases, including dbSNP (https://www.ncbi.nlm.nih.gov/SNP), ExAC (exac. Broad institute.org), 1000 genomes (http://www.international genome.org/), gnomAD (gnomad.broadinstitute.org/), Iranome (www.iranome.ir), and in-house database of WES was very low. PolyPhen-2 and SIFT, MutationTaster, and UMD-Predictor predicted that this variant was damaging, causes disease, and is pathogenic, respectively. According to the crystal structure of LG4 domain (pdb 5IK8), protein modeling showed that the p.G2889 is a buried amino acid in the 3D structure of the protein, which is conserved among different species (Fig. 1A), as well as human LG domains (Fig. 1B)[23]. The C- terminus of *LAMA2* consists of five laminin G domains that form the globular part of the laminin, which engage in network formation and cell-surface interaction[21,24,25]. The modeling result showed that the substitution of Arg at position 2889 may affect the α2 laminin G4 structure and calcium-binding site, resulting in the disruption of binding site for heparin and dystroglycan molecules (Fig. 2). The result also suggested that this mutation may be a common mutation among Iranian patients, and/or it may show ancestral relationship among MCDA1 patients. Therefore, evidence mentioned above suggested c.8665G>A as a variant of pathogenic mutation.

**Fig. 3:**
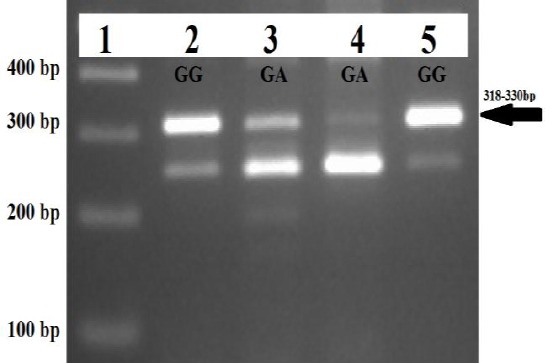
The result of the partial *LAMA2*-cDNA analysis for the c.7452-1G>A splicing mutation. Lane 1, a 100-bp DNA ladder; Lane 5, *LAMA2* normal control with wild-type genotype GG for c.7452-1G>A variant. Arrow shows normal partial *LAMA2*-cDNA with the product size of 318-330 bp. Lane 2 (father) has a wild-type genotype GG, while both lane 3 (mother) and lane 4 (patient) have heterozygotes genotype GA for c.7452-1G>A mutation. The arrow shows normal partial *LAMA2*-cDNA with the product size of 318-330 bp.

In patient 2, the c.397-4_c.478del splicing mutation is an 86-bp deletion at the DNA level that may cause the skipping of exon-4 or nonsense-mediated mRNA decay, resulting in the disruption of the laminin N-terminal domain (LamNT, domain VI) or the truncated protein. The subsequent RT-PCR analysis suggested that c.7452-1G>A, in patient 2, may cause the skipping of exon-54 (Fig. 3), which affects the α2 laminin G2 domain. This mutation along with c.397-4_c.478del together with c.397-4_c.478del caused the disease in patient 2. Compound heterozygous mutation has previously been reported in a severe form of CMD[26].

Regarding the analysis of known genes in CMDs, we can conclude that WES can be more beneficial and cost-effective than direct sequencing of large genes or NGS-targeted panels for identification of causal variants. The finding of this study demonstrated that the identified mutations were consistent with the diagnosis of MDC1A. The disease is very rare in Iran, and more studies are needed to provide extensive information about the mutation spectra of the *LAMA2* gene in this country. Our finding would be helpful in diagnosis, genetic counseling, and prenatal diagnosis.
